# Supplementary material for: Association of the use of hearing aids with the conversion from mild cognitive impairment to dementia and progression of dementia: A longitudinal retrospective study
Source: Alzheimers Dement (N Y). 2021 Feb 14;7(1):e12122. doi: 10.1002/trc2.12122 (PMC7882528; doi:10.1002/trc2.12122)
Supplement: Supplementary file 1 — Supplementary information [file TRC2-7-e12122-s001.docx]

**Table S1.** Baseline demographic and clinical characteristics of participants included and excluded from the study.

|  | **Group 1**  MCI at baseline | |  | **Group 2**  Dementia at baseline | |  |
| --- | --- | --- | --- | --- | --- | --- |
|  | Excluded | Included |  | Excluded | Included |  |
| Total | 4461 | 939 |  | 8951 | 1175 |  |
| Gender, No. (%) |  |  |  |  |  |  |
| Male | 2055 (46.1) | 578 (61.6) | < 0.001 | 4259 (47.6) | 757 (64.4) | < 0.001 |
| Female | 2406 (53.9) | 361 (38.4) |  | 4692 (52.4) | 418 (35.6) |  |
| Age, mean (SD), years | 74.7 (9.0) | 78.9 (8.0) | < 0.001 | 73.4 (9.6) | 78.0 (8.5) | < 0.001 |
| Education, mean (SD), years^a^ | 15.5 (5.9) | 16.1 (7.4) | 0.03 | 15.3 (8.3) | 15.1 (3.5) | 0.12 |
| CDR-SB score, mean (SD) | 1.4 (1.1) | 1.5 (1.2) | 0.01 | 5.7 (3.9) | 5.4 (3.7) | 0.01 |

Abbreviation: SD, standard deviation; CDR-SB, Clinical Dementia Rating Sum of Boxes

^a^ measured as the number of years of education completed

**Table S2.** Sensitivity analysis based on the inverse probability weighting

| Characteristic | Level | Total | Hazard Ratio (95% CI) | | *P* value | | FDR *P* value | |
| --- | --- | --- | --- | --- | --- | --- | --- | --- |
| *Group 1* | | | |  | |  | |  |
| Hearing Aid | No | 303 |  | |  | |  | |
|  | Yes | 636 | 0.72 (0.60, 0.88) | | <0.001 | | 0.003 | |
| Gender | Male | 578 |  | |  | |  | |
|  | Female | 361 | 0.98 (0.81, 1.20) | | 0.87 | | 0.87 | |
| Age |  | 939 | 1.01 (0.99, 1.02) | | 0.31 | | 0.52 | |
| Education |  | 939 | 1.00 (0.98, 1.01) | | 0.69 | | 0.86 | |
| CDR-SB score |  | 939 | 1.35 (1.25, 1.46) | | < 0.001 | | < 0.001 | |
| *Group 2* | |  |  | |  | |  | |
| Hearing Aid | No | 465 |  | |  | |  | |
|  | Yes | 710 | 0.99 (0.80, 1.23) | | 0.92 | | 0.92 | |
| Gender | Male | 757 |  | |  | |  | |
|  | Female | 418 | 0.73 (0.60, 0.91) | | 0.004 | | 0.01 | |
| Age |  | 1175 | 1.02 (1.00, 1.03) | | 0.01 | | 0.02 | |
| Education |  | 1175 | 1.01 (0.98, 1.04) | | 0.49 | | 0.62 | |
| CDR-SB score |  | 1175 | 1.08 (1.05, 1.10) | | < 0.001 | | < 0.001 | |

Abbreviation: CDR-SB: Clinical Dementia Rating Sum of Boxes; FDR: false discovery rate.

**Table S3.** Risk of incident all-cause dementia (Group 1) and risk of death (Group 2) by hearing aid status. The group of hearing aid users consisted of subjects with functionally normal hearing with a device and those that exhibited reduced ability to do everyday activities such as listening to the radio/television or talking with family/friends when wearing a hearing aid

| Characteristic | Level | Total | Hazard Ratio (95% CI) | | *P* value | | FDR *P* value | |
| --- | --- | --- | --- | --- | --- | --- | --- | --- |
| *Group 1* | | | |  | |  | |  |
| Hearing Aid | No | 303 |  | |  | |  | |
|  | Yes | 765 | 0.74 (0.61, 0.89) | | 0.001 | | 0.003 | |
| Gender | Male | 659 |  | |  | |  | |
|  | Female | 409 | 1.05 (0.89, 1.25) | | 0.55 | | 0.69 | |
| Age |  | 1068 | 1.01 (0.99, 1.02) | | 0.32 | | 0.54 | |
| Education |  | 1068 | 1.00 (0.99, 1.01) | | 0.82 | | 0.82 | |
| CDR-SB score |  | 1068 | 1.40 (1.32, 1.49) | | < 0.001 | | < 0.001 | |
| *Group 2* | |  |  | |  | |  | |
| Hearing Aid | No | 465 |  | |  | |  | |
|  | Yes | 884 | 0.99 (0.80, 1.23) | | 0.92 | | 0.89 | |
| Gender | Male | 876 |  | |  | |  | |
|  | Female | 473 | 0.73 (0.60, 0.91) | | 0.004 | | 0.01 | |
| Age |  | 1349 | 1.02 (1.00, 1.03) | | 0.01 | | 0.02 | |
| Education |  | 1349 | 1.01 (0.98, 1.04) | | 0.49 | | 0.61 | |
| CDR-SB score |  | 1349 | 1.07 (1.05, 1.10) | | < 0.001 | | < 0.001 | |

Abbreviation: CDR-SB: Clinical Dementia Rating Sum of Boxes; FDR: false discovery rate.
